# High-Salt Diet in the Pre- and Postweaning Periods Leads to Amygdala Oxidative Stress and Changes in Locomotion and Anxiety-Like Behaviors of Male Wistar Rats

**DOI:** 10.3389/fnbeh.2021.779080

**Published:** 2022-01-04

**Authors:** Pedro Ernesto de Pinho Tavares Leal, Alexandre Alves da Silva, Arthur Rocha-Gomes, Tania Regina Riul, Rennan Augusto Cunha, Christoph Reichetzeder, Daniel Campos Villela

**Affiliations:** ^1^Programa de Pós-Graduação em Ciências da Saúde, Universidade Federal dos Vales do Jequitinhonha e Mucuri, Diamantina, Brazil; ^2^Laboratório de Nutrição Experimental – LabNutrex, Departamento de Nutrição, Universidade Federal dos Vales do Jequitinhonha e Mucuri, Diamantina, Brazil; ^3^Programa de Pós-Graduação em Ciências da Nutrição, Universidade Federal dos Vales do Jequitinhonha e Mucuri, Diamantina, Brazil; ^4^Department of Nutritional Toxicology, Institute of Nutritional Science, University of Potsdam, Potsdam, Germany

**Keywords:** high-sodium, open-field, elevated plus-maze, pre-natal, post-natal, redox state

## Abstract

High-salt (HS) diets have recently been linked to oxidative stress in the brain, a fact that may be a precursor to behavioral changes, such as those involving anxiety-like behavior. However, to the best of our knowledge, no study has evaluated the amygdala redox status after consuming a HS diet in the pre- or postweaning periods. This study aimed to evaluate the amygdala redox status and anxiety-like behaviors in adulthood, after inclusion of HS diet in two periods: preconception, gestation, and lactation (preweaning); and only after weaning (postweaning). Initially, 18 females and 9 male Wistar rats received a standard (*n* = 9 females and 4 males) or a HS diet (*n* = 9 females and 5 males) for 120 days. After mating, females continued to receive the aforementioned diets during gestation and lactation. Weaning occurred at 21-day-old Wistar rats and the male offspring were subdivided: control-control (C-C)—offspring of standard diet fed dams who received a standard diet after weaning (*n* = 9–11), control-HS (C-HS)—offspring of standard diet fed dams who received a HS diet after weaning (*n* = 9–11), HS-C—offspring of HS diet fed dams who received a standard diet after weaning (*n* = 9–11), and HS-HS—offspring of HS diet fed dams who received a HS diet after weaning (*n* = 9–11). At adulthood, the male offspring performed the elevated plus maze and open field tests. At 152-day-old Wistar rats, the offspring were euthanized and the amygdala was removed for redox state analysis. The HS-HS group showed higher locomotion and rearing frequency in the open field test. These results indicate that this group developed hyperactivity. The C-HS group had a higher ratio of entries and time spent in the open arms of the elevated plus maze test in addition to a higher head-dipping frequency. These results suggest less anxiety-like behaviors. In the analysis of the redox state, less activity of antioxidant enzymes and higher levels of the thiobarbituric acid reactive substances (TBARS) in the amygdala were shown in the amygdala of animals that received a high-salt diet regardless of the period (pre- or postweaning). In conclusion, the high-salt diet promoted hyperactivity when administered in the pre- and postweaning periods. In animals that received only in the postweaning period, the addition of salt induced a reduction in anxiety-like behaviors. Also, regardless of the period, salt provided amygdala oxidative stress, which may be linked to the observed behaviors.

## Introduction

Sodium chloride (NaCl), also known worldwide as salt, is one of the most widely used condiments in food processing (Steffensen et al., 2018). It is estimated that current salt intake averages are 6 g/day in most countries (86% greater than the optimal amount), with varying usages ranging from food preservation to flavor enhancement (Afshin et al., 2019; Tan et al., 2021). Excessive use of salt in the diet is responsible for the development mainly of cardiovascular diseases (Huang et al., 2020; Neal et al., 2021), but also stomach cancer (Ge et al., 2012), kidney diseases (Garofalo et al., 2018), and osteoporosis (Fatahi et al., 2018). Moreover, recent data indicates that high-salt diets were directly related to approximately three million deaths in 1 year, being classified as one of the top 3 dietary risk factors for health (Bill and Foundation, 2019; He et al., 2020).

In addition to the known harmful health effects, the use of high-salt diets has recently been linked to cerebrovascular diseases and cognitive impairment in humans (Heye et al., 2016). Studies in rodents that used dietary or water salt supplementation (2–8%) confirm these findings, reporting impaired cognition, aggravation of cerebral ischemic injury, and high-stress responsivity (Ge et al., 2017; Faraco et al., 2018, 2019; Mitchell et al., 2018; Gilman et al., 2019a; Zhang et al., 2020). Importantly, preclinical studies suggest that the maternal high-salt diet can also induce changes in locomotion, inhibition, and anxiety in the offspring, when fed in the preconception, gestation, or lactation periods (Mcbride et al., 2008; Mecawi and Almeida, 2017; Dingess et al., 2018). During these periods, the offspring is highly susceptible to dietary salt, which may impact on development, potentially leading to lifelong changes in metabolism and behavior. These changes are related to the Developmental Origin of Health and Disease (DOHaD), which proposes that adversities in early life can result in persistent changes in physiology, leading to an increased risk of developing diseases in adulthood (O’Donnell and Meaney, 2016; Klein et al., 2018; de Souza et al., 2020a).

One of the main possible mechanisms for behavioral changes caused by salt consumption is related to the oxidative stress (Santisteban and Iadecola, 2018; He et al., 2020). Evidence indicates that a high-salt diet can reduce nitric oxide (NO) production (Dong et al., 2011; Kouyoumdzian et al., 2016; Zheng et al., 2019), suppress the activity of antioxidant enzymes (Kitiyakara et al., 2003; Huang et al., 2017), and increase the production of nitrogen and oxygen-free radicals (Kitiyakara et al., 2003; Huang et al., 2017; Zheng et al., 2019). Also, it is highlighted that a high-salt diet causes oxidative stress in the hippocampus, hypothalamus, and cerebellum, important brain regions for behavior and cognition (Bai et al., 2017; Ge et al., 2017; Stocher et al., 2018). However, to the best of our knowledge, there are no studies evaluating the amygdala redox status after administration of a high-salt diet, either before weaning (preweaning) or after weaning (postweaning). Noteworthy, the amygdala is a major brain region in the interpretation of environmental threats, possibly related to anxiety-like and fear behaviors in rodents (Calhoon and Tye, 2015; Wilson et al., 2015; dos Santos et al., 2017).

Therefore, this study aimed to evaluate the effects of the high-salt diet on amygdala redox status and anxiety-like behaviors at adulthood, considering: (1) the inclusion of the salt in the preconception, gestation, and lactation periods (preweaning) and (2) the addition of salt in the diet only after weaning until adulthood (postweaning). The main hypothesis was that the high-salt diet may result in amygdala oxidative stress regardless of the period, which, in turn, would promote changes in anxiety-like behaviors at adulthood.

## Materials and Methods

### Ethics

This experimental protocol was approved by the Ethics Committee on the Use of Animals of Universidade Federal dos Vales do Jequitinhonha e Mucuri (CEUA-UFVJM) (protocol 025/2018). These are also in agreement to the ethical principles of the National Institutes of Health Guide for the Care and Use of Laboratory Animals (NIH Publications No. 80-23). All the rats (Wistar—*Rattus norvegicus*) were obtained from Laboratório de Pós-Graduação e Pesquisa (LPP-UFVJM) and housed in conditions of natural moisture, temperature of 22 ± 2°C (controlled by an air conditioner), and a 12-h cycle of light and darkness, with the light cycle beginning at 7:00 am. All the animals had free access to potable water and their respective diets.

### Experimental Design

Initially, 18 female and 9 male Wistar rats aged 21 days were used. The animals were housed in 3 per box according to sex in order to randomly receive the diets for a duration of 120 days: **control (C)**: received standard diet (laboratory chow for rodents: Nuvilab^®^ CR-1, Quimtia S/A, Paraná, Brazil) (*n* = 9 females and 4 males) or **high-salt (HS) diet**: received laboratory chow with added salt (4% NaCl non-iodized, Mossoró^®^—purity 96.04% bought at the local store) (*n* = 9 females and 5 males). Copulation was evaluated every morning and confirmed by the presence of sperm in the vaginal smear, which was considered the beginning of gestation. All the animals received food and water *ad libitum*. After this period, the nulliparous female rats (141 days old) were placed for mating with males (1 male to 3 females) during the dark cycle (7:00 pm to 7:00 am) every day. Parents during mating (males and females) and dams during gestation and lactation continued receiving the aforementioned diets (control or HS). At birth, the litters were culled to eight pups (6 males and 2 females).

In the postweaning period, only male offspring were used, housed 3 animals per box. Male offspring was randomly allocated to receive either control (laboratory chow Nuvilab^®^ CR-1) or HS diets (laboratory chow with added salt 4% NaCl non-iodized). Therefore, the offspring were subdivided into the following groups: **control-control (C-C)**—offspring of standard diet fed dams who received a standard diet after weaning (*n* = 9–11), **control-high-salt (C-HS)**—offspring of standard diet fed dams who received a HS diet (laboratory chow with added salt at 4% NaCl non-iodized) after weaning (*n* = 9–11), **HS-C**—offspring of HS diet fed dams who received a standard diet after weaning (*n* = 9–11), and **HS-HS**—offspring of HS diet fed dams who received a HS diet (laboratory chow with added salt at 4% NaCl non-iodized) after weaning (*n* = 9–11).

**FIGURE 1:**
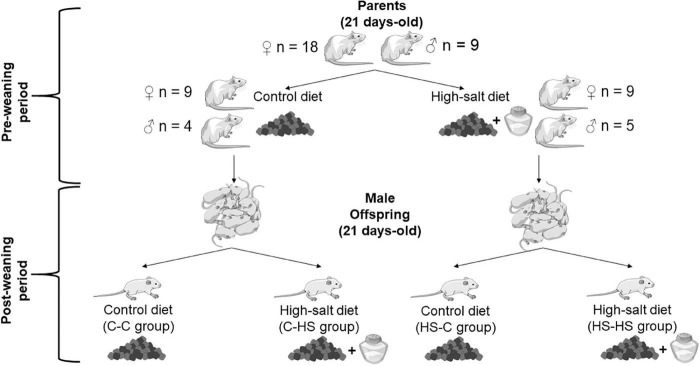
Representation of the experimental design. Control (standard diet; laboratory chow Nuvilab^®^ CR-1) or high-salt (HS) (laboratory chow with added salt 4% NaCl non-iodized) diets were provided for parents at 21 day-old. They received their respective diets for 120 days, until they reached adulthood. During the periods of mating, gestation, and lactation, the dams remained on the aforementioned diets. The preconception, gestation, and lactation periods were classified as “preweaning.” At 21 day-old, the offspring were weaned and received until adulthood the control or HS diets. The period from weaning to adulthood was classified as “postweaning.” Therefore, the offspring were subdivided into the groups: control-control (C-C), offspring of standard diet fed dams who received a standard diet after weaning; control-HS (C-HS), offspring of standard diet fed dams who received a HS diet after weaning; HS-C, offspring of HS diet fed dams who received a standard diet after weaning; and HS-HS, offspring of HS diet fed dams who received a HS diet after weaning.

The male offspring received the aforementioned diets until adulthood (141 day-old), when behavioral tests were carried out. Approximately, 1–2 animals from each litter were used for the behavioral and redox status analyses, in order to reduce litter effects. The experimental design is shown in Figure 1.

### Offspring Behavior

All the tests were performed in an isolated room (130 lux) and in a double-blind manner. The offspring performed the elevated plus maze (EPM) (141 day-old) and open field (OF) (151 day-old) tests, both during the morning period (7:00–12:00 am). A camera (Sony Handycam^®^) was positioned above the arena and two independent, blinded, and experienced studies later evaluated the randomly arranged videos. Between the performances of the two behavioral tests (EPM and OF), the animals were kept with their respective diets in the conditions mentioned previously. All the equipment used was cleaned with 70% ethanol between each test to eliminate olfactory cues.

The EPM test is based on the aversion to open and high spaces of the rodents and is a classic test for assessing anxiety-like behaviors (Pellow et al., 1985; de Souza et al., 2020b). The EPM is made of wood, with two closed arms (50 cm × 10 cm × 40 cm) perpendicular to two open arms (50 cm × 10 cm), besides a central area (10 cm × 10 cm), raised 50 cm high from the floor. Each rat was placed individually in the central area of the EPM with its head facing toward one closed arm and its movements were filmed for 10 min (Teixeira et al., 2020). The ratio of entries (considered as the animal inserting all the four paws) in each arm (closed or open) and the time spent in them were evaluated (Teixeira et al., 2020). In addition, to analyze the risk assessment of animal, the frequency of head-dipping (the head flexes below the edge of the open arms), rearing (frequency with which the animal stands on its hind legs), and grooming (frequency of time which the animal spent licking or scratching itself while stationary) was recorded (Plescia et al., 2015; Guedine et al., 2018; Riul and Almeida, 2020).

The OF test is widely used to check locomotion of animal through distance covered, but is also used to evaluate anxiety-like behaviors over the conflict between exploring a new environment and exposed to an open arena (Montgomery, 1955). The OF is a square wooden arena, with total dimensions of 70 cm × 70 cm × 50 cm (dimensions of central zone of the arena: 35 cm × 35 cm), being subdivided into 16 quadrants (17.5 cm × 17.5 cm). Each animal was placed in the center of the OF and free exploration was allowed for 10 min (Teixeira et al., 2020). The parameters of center zone entries frequency (defined when the animal inserted the four paws in the central zone), time spent in the center zone, distance covered (quadrants), rearing, and grooming frequency were observed (Teixeira et al., 2020; Rocha-Gomes et al., 2021a).

### Redox State

The animals were euthanized by decapitation when they were at 152 day-old. The whole brain was rapidly removed (<1 min) and submerged on cold (4°C) phosphate-buffered saline (PBS) (50 mM; pH 7.0), followed by the amygdala dissection (Paxinos and Watson, 2014). After, the tissues were homogenized in cold PBS (4°C; 50 mM; pH 7.0) and centrifuged at 750 × *g* for 10 min at 4°C (Melo et al., 2019). Both the sides of the amygdala were used for the analysis of the total antioxidant capacity, activity of antioxidant enzymes, and oxidative stress marker.

The total antioxidant capacity was evaluated using the ferric reducing antioxidant power (FRAP) method (Benzie and Strain, 1996). The assay is based on the ability of the antioxidant compounds of the sample to reduce the ferric-tripyridyltriazine complex to ferrous tripyridyltriazine, monitored at 550 nm. Ferrous sulfate (FeSO_4_) was used as standard and the results were reported as nM of FeSO_4_/mg protein (Freitas et al., 2019).

For the activity of the antioxidant enzyme superoxide dismutase (SOD), a solution containing 50 mM potassium dihydrogen phosphate (KH_2_PO_4_) and 1 mM diethylene-triamine-pentaacetic acid (DTPA) was added to the tissue homogenate. Following this, 0.2 mM of pyrogallol was added and its oxidation was measured at 420 nm for 250 s at interval of 10 s. The results were defined as one unit (U) of SOD per mg protein in the sample (U/mg protein) (Marklund and Marklund, 1974; Melo et al., 2019).

Catalase (CAT) activity was assessed by metabolizing hydrogen peroxide (Nelson and Kiesow, 1972). To perform this test, 5 μl of hydrogen peroxide (0.3 M) was added to a solution containing potassium phosphate buffer (50 mM; pH 7.0; 25°C) and 30 μl of sample. The readings were performed in a microplate reader every 15 s for 1 min (at 25°C). CAT activity was expressed in ΔE/min/mg of protein (Freitas et al., 2019).

Glutathione S-transferase (GST) activity was estimated spectrophotometrically as previously described (Habig et al., 1974). The assay occurred according to the formation of glutathione conjugated with 2,4-dinitrochlorobenzene (molar coefficient extinction: ε340 = 9.6 mmol × L^–1^ × cm^–1^). One unit of GST activity was defined as the amount of the enzyme that catalyzed the formation of one μmol of product × min^–1^ × mL^–1^ (Rocha-Gomes et al., 2021a).

The lipid peroxidation evaluation was performed using the thiobarbituric acid reactive substances (TBARS) method and is classified as an oxidative stress marker (Ohkawa et al., 1979). A solution containing acetic acid (2.5 M; pH 3.4), thiobarbituric acid (0.8%), and sodium dodecyl sulfate (8.1%) was added to the tissue sample for 90 min at 95°C. The TBARS formation was evaluated at 532 nm using malondialdehyde (MDA) (1,1,3,3-tetramethoxypropane) as the standard. The results are expressed in nmol MDA/mg protein (Freitas et al., 2019).

All the redox analyses were performed in triplicate using a plate reader (UV/Visible U-200 L Spectrophotometer). Protein content was quantified using bovine serum albumin (BSA) (1 mg/ml) as the standard (Bradford, 1976). The results of the redox state were corrected for the amount of protein in the samples.

### Statistical Analysis

Statistical analysis was performed with Statistica software (version 10.0, StatSoft^®^, Hamburg, Germany). Graphics were made using the GraphPad Prism^®^ version 7.0 (GraphPad, La Jolla, CA, United States). Sample normality was evaluated using the Shapiro–Wilk test. Data with normal distribution were analyzed using the two-way ANOVA, with the factors: preweaning (received standard or HS diets until weaning) and postweaning (received standard or HS diets only after weaning). The Newman–Keuls was used as a *post hoc* test when appropriate (*p* < 0.05). Data with non-normal distributions were analyzed by the Kruskal–Wallis test with the Dunn’s *post hoc* test. Results are expressed as a mean and SEM.

## Results

**FIGURE 2:**
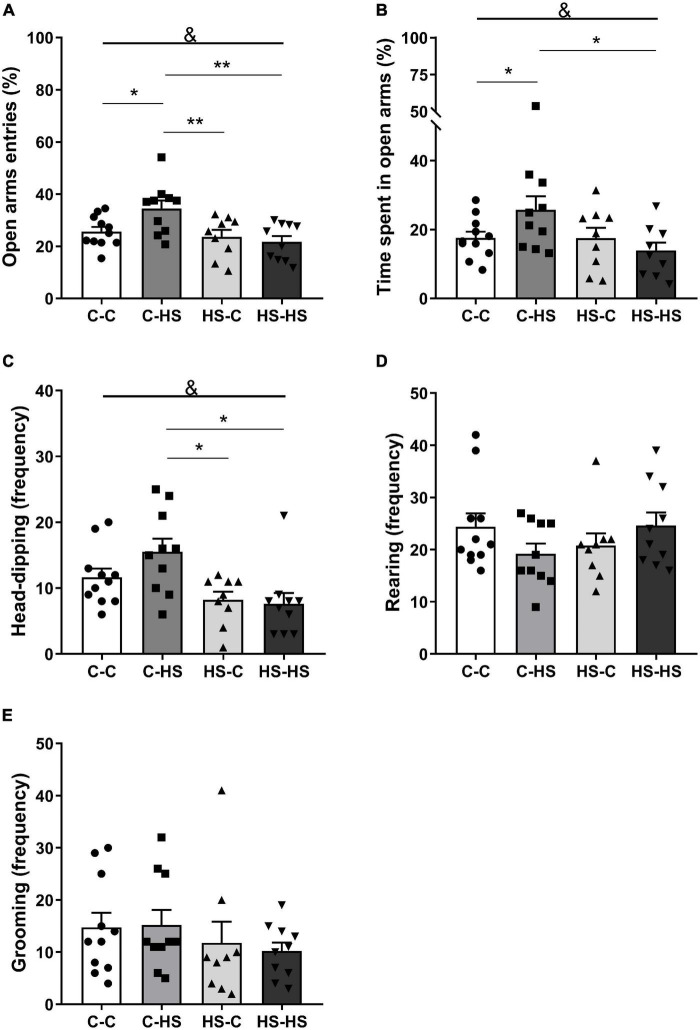
Ratio of entries **(A)** and time spent in the open arms **(B)**; head-dipping **(C)**, rearing **(D)**, and grooming **(E)** frequency in the elevated plus maze test. C-C, offspring of standard diet fed dams who received a standard diet after weaning; C-HS, offspring of standard diet fed dams who received a HS diet after weaning; HS-C, offspring of HS diet fed dams who received a standard diet after weaning; and HS-HS, offspring of HS diet fed dams who received a HS diet after weaning. Data are shown as mean and SEM; *n* = 9–11; &*p* < 0.05 (preweaning factor); *p < 0.05, **p < 0.01 (interaction of the pre- and postweaning factors) using the ANOVA and the Newman–Keuls tests.

In the EPM test, the ratio of entries in the open arms showed a significant difference in the preweaning factor [*F*_PRE (1_,_36)_ = 9.19, *p* < 0.01]. The offspring who received a HS diet until weaning entered less in the open arms compared to the offspring of standard diet fed dams (*p* < 0.01). In addition, an interaction in the factors pre- and postweaning was observed [*F*_PRE__×__POST (1_,_36)_ = 4.93, *p* < 0.05]. The C-HS group showed higher ratio of entries in the open arms compared to the C-C (*p* < 0.05), HS-C (*p* < 0.01), and HS-HS (*p* < 0.01) groups (Figure 2A). Similarly, the ratio of time spent in the open arms showed a difference in the preweaning factor [*F*_PRE (1_,_36)_ = 4.36, *p* < 0.05]. The offspring who received a HS diet until weaning spent less time in the open arms compared to the offspring of standard diet fed dams (*p* < 0.05). Also, an interaction in the factors pre- and postweaning was observed [*F*_PRE__×__POST (1_,_36)_ = 4.28, *p* < 0.05]. The C-HS group spent more time in the open arms in relation to the C-C (*p* < 0.05) and HS-HS (*p* < 0.05) groups (Figure 2B). For the head-dipping frequency, a significant difference in the preweaning factor could be seen [*F*_PRE (1_,_36)_ = 12.52, *p* < 0.01]. The offspring who received a HS diet until weaning showed lower head-dipping frequency compared to the offspring of standard diet fed dams (*p* < 0.01). Moreover, a difference in the interaction of pre- and postweaning factors was observed [*F*_PRE__×__POST (1_,_36)_ = 1.97, *p* < 0.05]. The C-HS group performed head-dipping more frequently compared to the HS-C (*p* < 0.01) and HS-HS (*p* < 0.01) groups (Figure 2C). No differences were found in the evaluation of rearing (*p* = 0.06) and grooming frequency (*p* = 0.73) in the EPM test (Figures 2D,E).

**FIGURE 3:**
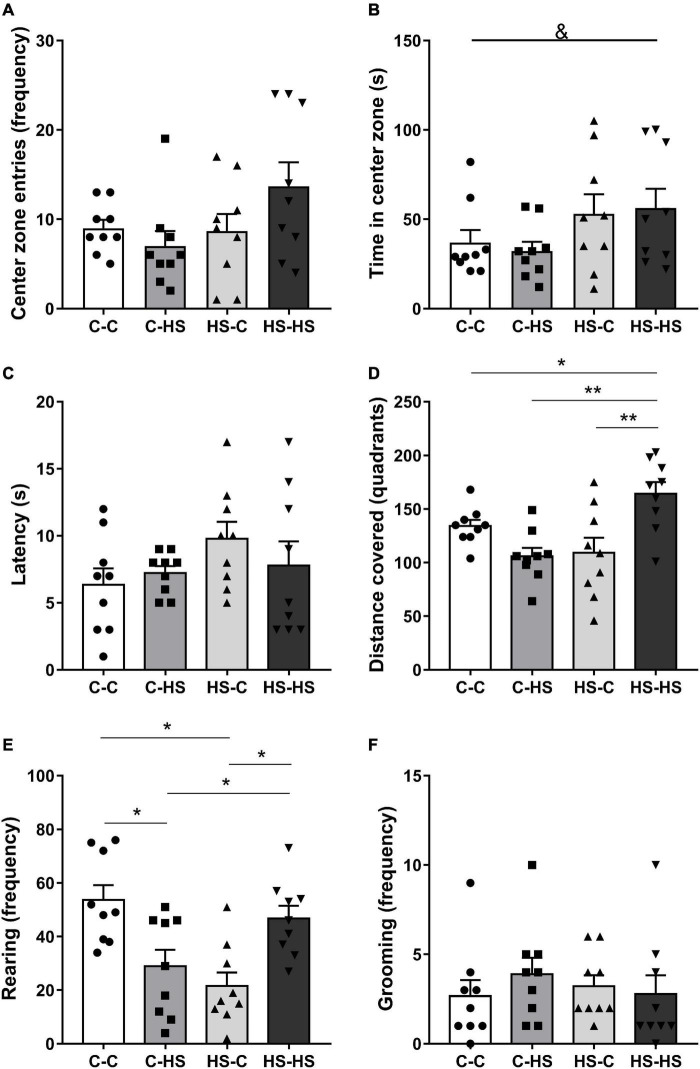
Entries frequency **(A)**, time spent at the central zone **(B)**, latency to leave the center zone **(C)**, distance covered **(D)**, rearing **(E)**, and grooming frequency **(F)** in the open field test. C-C, offspring of standard diet fed dams who received a standard diet after weaning; C-HS, offspring of standard diet fed dams who received a HS diet after weaning; HS-C, offspring of HS diet fed dams who received a standard diet after weaning; and HS-HS, offspring of HS diet fed dams who received a HS diet after weaning. Data are shown as mean and SEM; *n* = 9. &*p* < 0.05 (preweaning factor); *p < 0.05, **p < 0.01 (interaction of the pre- and postweaning factors) using the ANOVA and the Newman–Keuls tests.

In the evaluation of the time spent in the OF central zone, a difference was found with respect to the preweaning diet [*F*_PRE (1_,_32)_ = 5.12, *p* < 0.05]. The offspring who received a HS diet until weaning remained more time in the central zone of the OF test compared to the offspring of standard diet fed dams (*p* < 0.05) (Figure 3B). The total distance covered in the OF test showed a difference in the interaction of pre- and postweaning diets [*F*_PRE__×__POST (1_,_32)_ = 16.59, *p* < 0.01]. The HS-HS group reported higher locomotion in relation to the C-C (*p* < 0.05), C-HS (*p* < 0.01), and HS-C (*p* < 0.01) groups (Figure 3D). The rearing frequency in the OF test showed a difference in the interaction of pre- and postweaning diets [*F*_PRE__×__POST (1_,_32)_ = 0.16, *p* < 0.05]. The C-HS and HS-C groups accomplished lower numbers of rearing in relation to the C-C and HS-HS groups (*p* < 0.05) (Figure 3E). No differences were shown in the evaluation of latency to escape of center zone (*p* = 0.27) and grooming frequency (*p* = 0.35) in the OF test (Figures 3A,C,F).

**FIGURE 4:**
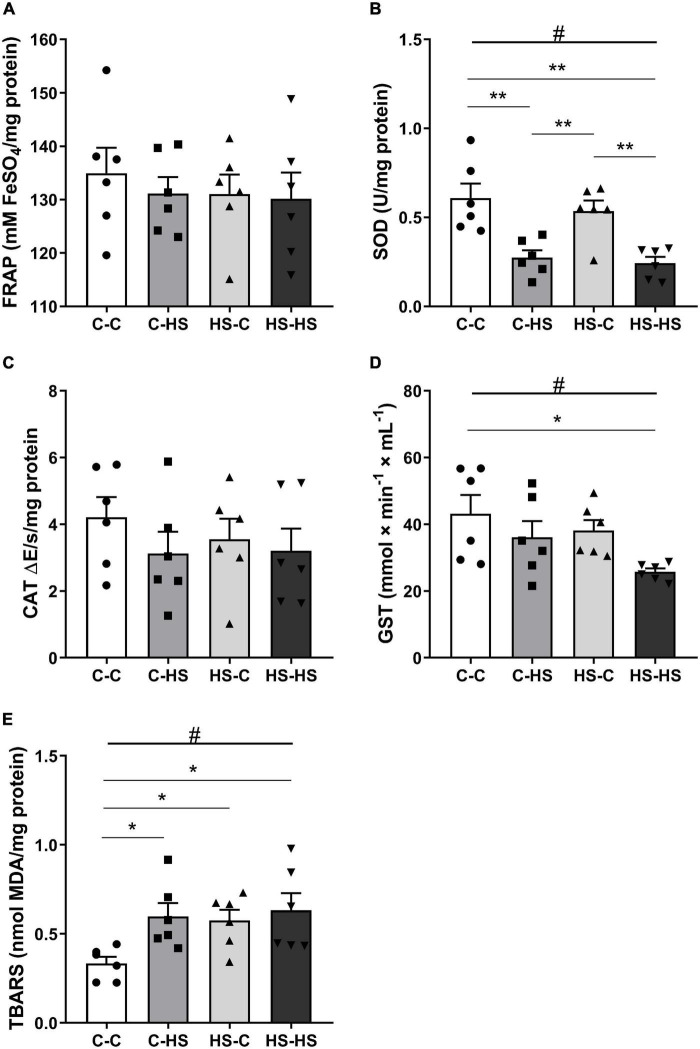
The ferric reducing antioxidant power (FRAP) total antioxidant capacity **(A)**, superoxide dismutase (SOD) **(B)**, catalase (CAT) **(C)**, glutathione S-transferase (GST) activity **(D)**, and the thiobarbituric acid reactive substances (TBARS) **(E)** concentration in the amygdala. C-C, offspring of standard diet fed dams who received a standard diet after weaning; C-HS, offspring of standard diet fed dams who received a HS diet after weaning; HS-C, offspring of HS diet fed dams who received a standard diet after weaning; and HS-HS, offspring of HS diet fed dams who received a HS diet after weaning. Data are shown as mean and SEM; *n* = 6. #*p* < 0.05 (postweaning factor); *p < 0.05, **p < 0.01 (interaction of the pre- and postweaning factors) using the ANOVA and the Newman–Keuls tests.

In the amygdala redox state evaluation, a difference with respect to the postweaning diet factor was shown for SOD analysis [*F*_POST (1_,_20)_ = 29.92, *p* < 0.001]. The offspring who received a HS diet after weaning reported less SOD activity compared to the offspring of standard diet fed dams (*p* < 0.001). In addition, an interaction in the pre- and postweaning diets was observed [*F*_PRE__×__POST (1_,_20)_ = 0.13, *p* < 0.05]. The C-HS and HS-HS groups showed less SOD activity compared to the C-C and HS-C groups (*p* < 0.01) (Figure 4B). For GST activity, a difference was shown in the postweaning diet [*F*_POST (1_,_20)_ = 5.69, *p* < 0.05]. The offspring who received a HS diet after weaning displayed less GST activity compared to the offspring of standard diet fed dams (*p* < 0.05). Also, an interaction in the pre- and postweaning diets was found [*F*_PRE__×__POST (1_,_20)_ = 0.42, *p* < 0.05]. The HS-HS group reported less GST activity with respect to the C-C group (Figure 4D). In the TBARS evaluation, a difference in the postweaning diet was observed [*F*_POST (1_,_20)_ = 5.21, *p* < 0.05]. The offspring who received a HS diet after weaning reported higher TBARS compared to the offspring of standard diet fed dams (*p* < 0.05). Moreover, an interaction of pre- and postweaning diets was observed [*F*_PRE__×__POST (1_,_20)_ = 2.14, *p* < 0.05]. The C-HS, HS-C, and HS-HS groups showed the higher TBARS levels compared to the C-C group (*p* < 0.05) (Figure 4E). No differences were reported in the FRAP (*p* = 0.16) and CAT (*p* = 0.57) evaluations (Figures 4A,C).

## Discussion

High-salt diets are consumed worldwide and are associated with cardiovascular morbidity and mortality. Noteworthy, HS intake has also been linked to behavioral changes in rodents. This study evaluated differential effects of HS and standard diet combinations given in the pre- or postweaning period. In this study, an increase in locomotion was showed in the group of animals that received a HS diet in, both, the pre- and postweaning period (HS-HS group). In addition, animals that received the HS diet only after weaning displayed a decrease in anxiety-like behaviors (C-HS group). Furthermore, both the groups showed amygdala oxidative stress, which may explain the behavioral changes observed.

The HS-HS group received the HS diet in both the periods (pre- and postweaning) resulting in adulthood hyperactivity measured by higher locomotion in the OF test. Also, this group presented an increase in the rearing frequency, which can be classified as a vertical exploration, confirming a high activity (Borta and Schwarting, 2005; Wardwell et al., 2020). Interestingly, with a similar protocol, Mcbride et al. (2008) observed that both the male Wistar rats treated with HS diet (4% NaCl) in the pre- (preconception and gestation periods) and postnatal periods (lactation) had increased locomotion in the OF test. In combination, these data indicate that a HS diet can induce hyperactivity in rodents. Moreover, these animals were more sensitive to the stimulating effect on locomotion produced by the administration of amphetamine compared to the group that received a standard diet (Mcbride et al., 2008). This result leads to the assumption that a HS diet of this study could also sensitize offspring to the effects of amphetamines. Interestingly, we have previously showed that cafeteria or calorie-restricted diets during lactation and postlactation can alter anxiety and locomotion of offspring after ephedrine (psychostimulant drug) application, reaffirming the role of diets in sensitization to some drugs by mechanisms that are not yet clearly established (Rocha-Gomes et al., 2021b).

Curiously, the spontaneously hypertensive rats (SHR) model consistently exhibits hyperactivity in the OF test (Botanas et al., 2016; Aparicio et al., 2019; Chen et al., 2019). This model was initially developed for the study of deleterious effects of cardiovascular diseases. However, due to its behavioral characteristics of hyperactivity, high impulsivity, and learning disabilities, SHR rats are also used as a model of attention-deficit/hyperactivity disorder (ADHD) (Leffa et al., 2019). It is important to note that the excessive salt consumption is recognized as a risk factor for the development of arterial hypertension (Valenzuela et al., 2021). In addition, rodents on HS diets during the pre- or postnatal periods can develop hypertension in adulthood (Contreras et al., 2000; Swenson et al., 2004). Although we did not use the SHR model in this study and did not check the blood pressure of animals, we speculated in relation to the similarities between the results presented by the HS-HS group and the SHR model. It is possible that a HS diet in the HS-HS group has programmed the mechanisms for controlling blood pressure and also induced hyperactive behavior in adulthood, similar to that observed in the studies with the SHR model. Therefore, a hyperactivity phenotype is suggested for the HS-HS group. However, further studies are needed to assess whether the phenotype presented by this group may have any relation to ADHD.

Furthermore, one of our main hypotheses was that a HS diet could promote changes in anxiety-like behavior at adulthood. In this study, the C-HS group reported less anxiety-like behavior in the EPM test, due to the higher ratio of entries and time spent in the open arms, in addition to the higher head-dipping frequency (Souto et al., 2020). Gilman et al. (2019a) observed that after a short exposure to a HS diet (4% NaCl; during 7 days), rodents reduced behavioral inhibition under relatively low-threat conditions. In particular, this means that a HS diet can decrease anxiety-like behavior in situations that would be naturally aversive to rodents, as in the EPM test. This has important implications; as by exposing themselves to open or higher spaces, these animals may be more exposed to risky conditions or even increasing their visibility to predators (Gilman et al., 2019a). This result of a higher activity in potentially aversive situations was found in male mice (C57BL/6J) using other paradigms after consuming a HS diet (4% NaCl; during 7 days) such as the forced swim test (Mitchell et al., 2018; Gilman et al., 2019b). In addition, the abovementioned studies observed amygdala inflammation (Mitchell et al., 2018; Gilman et al., 2019b), possibly establishing a link to a HS intake, low anxiety-like behavior, and cellular damages in a specific brain region. Although we cannot distinguish anxiety-like from impulsive behaviors, increased exploratory (horizontal and vertical) activity in new environments is a characteristic of impulsive behavior, which may also be caused by alterations in specific brain areas related to decision-making in adverse situations (Almeida et al., 1993). However, the reasons why the C-HS group had a lower frequency of rearing in the OF and a lower tendency in the EPM (with no statistical difference) tests remain to be clarified in future studies.

It is well established that experiences of mother during preweaning periods can modify the developmental health trajectory of her offspring. However, in some cases, no significant deleterious effects are observed, as demonstrated in the EPM test by the groups that received a HS diet in the preweaning period (HS-C and HS-HS). These observations are combined with the Predictive Adaptive Response (PAR) hypothesis, which argues that some changes that occur in early life in response to aversive stimuli are important to provide an advantage later in life. The PAR hypothesis predicts that these changes occur through epigenetic programming, which may also bring specific costs in the adult environment, making the animal maladapted on certain occasions (Raubenheimer et al., 2012; St-Cyr and McGowan, 2018). However, further studies are suggested to assess epigenetic changes that may be related to the results obtained here.

Reactive oxygen and nitrogen species can be considered as essential for the full development of neuronal functions when occurring in low or moderate amounts. However, at excessive levels, they are harmful and can lead to oxidative/nitrosative stress, causing damage to proteins, lipids, and nucleic acids (da Silva et al., 2014; Salim, 2017). In turn, this can lead to the release of inflammatory signals, resulting in neuroinflammation, loss of function, and, consequently, in behavioral changes (Hatanaka et al., 2016; Cirulli et al., 2020; Dias et al., 2020; Maciel August et al., 2020). Previous studies in rodents have shown that HS diets caused an imbalance in the brain redox state, with decreased cognition (Liu et al., 2014; Ge et al., 2017; Faraco et al., 2019) and increased reactivity to stressful situations (Bai et al., 2017; Dingess et al., 2018). Moreover, a HS diet in the preconception, gestation, and lactation periods has been shown to negatively influence the redox state of the cerebellum, hypothalamus, and hippocampus of the offspring (Stocher et al., 2018). These findings indicate a role of salt-rich diets with respect to the brain redox status, being able to induce oxidative stress in regions of fundamental importance for behavior and cognition.

The brain is very vulnerable to the excessive reactive oxygen and nitrogen species production, due to its high O_2_ consumption and modest antioxidant defenses (Bakunina et al., 2015; Salim, 2017). In addition, regions such as the hippocampus and the amygdala have been reported as the most susceptible to oxidative stress, consequently being more prone to functional decline (Bouayed et al., 2009; Salim, 2017). In this study, amygdala oxidative stress was observed, due to high levels of the TBARS (C-HS, HS-C, and HS-HS groups), in addition to the low activity of SOD (C-HS and HS-HS groups) and GST (HS-HS group) antioxidant enzymes. It is important to note that the amygdala plays a key role in the interpretation of environmental threats. Sensory stimuli are received in the amygdala that imbues them with emotional value and processing the outcomes as negative or positive valence, directly influencing anxiety-like behaviors mainly through the serotonergic system (Calhoon and Tye, 2015; dos Santos et al., 2017; de Lima et al., 2020). It is possible that diet-associated amygdala oxidative stress may be related to the behavioral alterations observed in the EPM and the OF tests; however, no clear patterns linking behavioral and redox readouts were noticeable in this study. Future studies are needed to better characterize this hypothetical relationship by also analyzing potential mediators that could serve as a link between changes in amygdala redox status and behavior.

In relation to the mechanism by which a HS diet can trigger oxidative stress of brain tissues, some suggestions based on previously published data are raised. The nuclear factor erythroid 2-related factor 2 (Nrf2) is a transcription factor that regulates the expression of several proteins, among them some involved in antioxidant defense system of cells. For example, antioxidant enzymes such as CAT, SOD, and GST are produced after activating the Nrf2 pathway (Iranshahy et al., 2018; Liu et al., 2020). Previously, Liu et al. (2020) showed a downregulation of the Nrf2 expression in renal tissue of rats receiving a HS diet. Similarly, Wang et al. (2020) reported high levels of reactive oxygen species and low activity of SOD and CAT in the hippocampus of HS diet rats. This result indicates that the downregulation of the Nrf2 pathway can occur not only at the systemic level, but also in the brain after consuming a HS diet. In addition, a HS diet can provide a reduction in NO production (Kouyoumdzian et al., 2016; Zheng et al., 2019). In situations where there is oxidative stress of the tissue, reactive oxygen species can inactivate NO (NO + O2^–^ → ONOO^–^). The radical ONOO^–^ is a very powerful oxidant and nitrosating agent. Thus, besides generates a toxic molecule (ONOO^–^), this reaction decreases the NO availability. NO plays an important role as a vasodilator, thus reducing it also contributing to arterial hypertension (Modlinger et al., 2004; Vaziri and Rodríguez-Iturbe, 2006). Notably, hypertension is strongly linked to oxidative stress (González et al., 2014; Ahmad et al., 2017; Guzik and Touyz, 2017; Small et al., 2018).

This study has some limitations. First, to better understand the relationship between amygdala oxidative stress and observed behavioral changes, it is necessary in the future the use of drugs that alter the production of reactive oxygen and nitrogen species and the evaluation of the Nrf2 expression. Second, the assessment of inflammation in the amygdala would be important to understand the real impact of a HS diet at the cellular level and the extent of tissue damage. Also, it is also important to evaluate serotonin levels in this brain region, since its concentration in the amygdala is directly related to anxiety-like behaviors. Third, the use of females is necessary, since sexual dimorphism is common in behavioral assessment studies. Females could have different responses due to other developmental vulnerabilities, altered neuroendocrine regulation, or placental and epigenetic different effects. Fourth, the evaluation of other tests related to anxiety-like (light-dark box and hole-board tests) and hyperactivity (SHR model; use of drugs that affect locomotion) behaviors must be performed to better understand the outcomes of this model. Fifth, finally, the next studies should assess blood pressure and heart rate, in an attempt to establish a link between these physiological responses and the observed behaviors.

In summary, this study demonstrated negative effects of a HS diet on the amygdala redox state. In addition, a HS diet promoted hyperactivity when administered in the combination of pre- and postweaning periods and decreased anxiety-like behaviors when offered only in the postweaning period. To the best of our knowledge, this is the first study that indicates damage to the amygdala in addition to behavior changes, regardless of the period in which salt is added to the diet. This fact is highlighted, due to the large consumption of salt in the world (Steffensen et al., 2018), its relationship with the development of cardiovascular diseases (Bill and Foundation, 2019; He et al., 2020), and with the hypotheses of behavioral changes and cognitive deficits after HS consumption also in humans (Heye et al., 2016; Abdoli, 2017; Afroz and Alviña, 2019).

## Conclusion

A HS diet promoted hyperactivity when administered in the pre- and postweaning periods. In animals that received only in the postweaning period, the addition of salt induced a reduction in anxiety-like behaviors. Regardless of the administration period, salt provided amygdala oxidative stress, which may be linked to the observed behaviors.

## Data Availability Statement

The original contributions presented in the study are included in the article/supplementary material, further inquiries can be directed to the corresponding author/s.

## Ethics Statement

The animal study was reviewed and approved by Ethics Committee on the Use of Animals of Universidade Federal dos Vales do Jequitinhonha e Mucuri (CEUA-UFVJM; protocol 025/2018).

## Author Contributions

All authors listed have made a substantial, direct, and intellectual contribution to the work, and approved it for publication.

## Conflict of Interest

The authors declare that the research was conducted in the absence of any commercial or financial relationships that could be construed as a potential conflict of interest.

## Publisher’s Note

All claims expressed in this article are solely those of the authors and do not necessarily represent those of their affiliated organizations, or those of the publisher, the editors and the reviewers. Any product that may be evaluated in this article, or claim that may be made by its manufacturer, is not guaranteed or endorsed by the publisher.
